# Computing Biology

**DOI:** 10.1371/journal.pcbi.1005050

**Published:** 2016-07-12

**Authors:** Ruth Nussinov, Jason A. Papin

**Affiliations:** 1 Cancer and Inflammation Program, Leidos Biomedical Research, Inc., Frederick National Laboratory for Cancer Research, National Cancer Institute, Frederick, Maryland, United States of America; 2 Sackler Institute of Molecular Medicine, Department of Human Genetics and Molecular Medicine, Sackler School of Medicine, Tel Aviv University, Tel Aviv, Israel; 3 Department of Biomedical Engineering, University of Virginia, Charlottesville, Virginia, United States of America

Is Computational Biology increasingly—and steadily—progressing toward addressing the mammoth challenge of actually *computing* biology? That is, have we reached the stage where we do not *support* biological research but *drive* it? This question is vitally important for all—young and established computational biologists. Even though forecasting future research can be risky, we still venture to predict that the future will see considerably more research projects drifting toward this ambitious aspiration. Computational Biology is powerful for abstracting signatures of disease, for predicting it, and for proposing medications. It is effective in figuring out disease mechanisms and forceful in bridging experimental disciplines to obtain testable predictions. However, perhaps its biggest challenges lie in putting together the available broad and disparate information, devising tools to efficiently and effectively carry out these tasks while sifting through noise and recognizing cell specificity, and most importantly coming up with sound, coherent, and testable schemes.

Among the examples of the complexity and the type of questions that we will increasingly face are the vast potential implications of findings that underscore the role of commensal microbiota in disease treatments. Further, the mechanisms which are involved—on the molecular level—are not understood and neither do we fully understand in detail how pathogens can modulate the host immune response and subvert it to their own advantage. We would like to identify—and understand—signatures of chronic inflammatory diseases; recurrent viral sequences in multiple patients and multiple cancers; we would like to map disease risks and to figure out what are the mechanisms for the distinct signaling of specific isoforms of oncogenic proteins in specific cancers. Analyses of cancer genomes points to driver mutations; however, we are baffled by the higher frequencies of specific mutations in certain tissues. We are also mystified by the complexity of the cellular network and its apparent redundancy which results in drug resistance. These are mere examples demonstrating the enormity of the questions which are facing us. Perhaps most tellingly is the fact that we are often even struggling to articulate specific objectives, to delineate the available knowledge and to evaluate apparent successes.

Within the grand challenge to compute biology, efforts to compute human health fosters multidirectional and multidisciplinary integration of basic, patient-oriented, and population-based research at different levels and across scales. Diverse data accumulate at increasingly high rates and quantities. We do not have a crystal ball for the future of research; we expect, however, that human health is going to be at the center. Human health is complex and encompasses multiple areas; thus, focusing on human health does not necessarily imply a narrower direction. Indeed, we expect that it will drive expansion, algorithm and tool development, and integration, all merging with experiments. Tools involving pattern recognition in images may receive a boost as well as those geared toward diverse, high volume computing. And most of all is the hurdle of making sense of observations to obtain coherent stories that would lead to new questions and new developments.

This shift toward computing human health has been in the making for some time. It reflects the huge accumulating data as well as the availability of increasingly powerful computing; it also reflects methodological advancements over a range of resolution scales. With new discoveries related to human health made almost every day and with these taken up by numerous labs, computational biology is faced with unprecedented challenges that we, as a community, must take up.

These are exciting times for Computational Biology. The field has expanded considerably; at the same time, its focus has been shifting. Areas once central to computational biology attract fewer investigators—often due to lack of sufficient funding as is the case with *ab initio* protein folding, where the experimental structural repertoire is growing and techniques such as electron microscopy are increasingly able to churn out structures at higher resolution. At the same time, other areas are gaining momentum and fast growing, particularly those related to human health. We believe that *PLOS Computational Biology* should not only capture these emerging trends but also promote and foster them. As the premier journal in Computational Biology *PLOS Computational Biology* is poised to lead and drive; not follow.

To be effective in continuing to lead the field requires an intense and well-planned coordinated effort, awareness and vigor. It requires familiarity with methodologies, types of data as they are becoming available, and initiatives to merge these together. It requires Focus Features, Perspectives and Reviews; it requires leadership and foresight in the increasingly diverse areas within these disciplines. We believe that to do this effectively, it requires a strong team at the helm. Doing it alone in addition to all the chores and travels may not achieve these aims at the level that we would like to see.

*PLOS Computational Biology* is our first and top priority and we have recognized a real need to ensure that overall leadership of the journal is strong, robust and experienced, along with the support of our Editorial Team and the community, to guide the journal through the next period of time. Until this date, Sebastian Bonhoeffer, Jason Papin, and Olaf Sporns have combined their talents and expertise to serve the journal at a very senior level as Deputy Editors-in-Chief. Olaf Sporns has taken on the Editorship of a new journal and so is stepping away from his Deputy Editor-in-Chief role on *PLOS Computational Biology*. He will, however, remain a member of our Editorial Advisory Board. In light of this development, and especially combined with the expanded breadth of our field, we wish to announce a new structure for leadership of the journal.

Following the successful model of other PLOS Community Journals and for the strength it will give to our journal, we are pleased to announce the appointment of Jason Papin as Co-Editor-in-Chief. We will share responsibility for the journal advancing it to meet the diverse emerging challenges. We are also very pleased to announce that Sebastian Bonhoeffer has agreed to assume the new position of Chair of the Editorial Advisory Board. In addition to continuing to serve as a senior level editor, Sebastian will begin to work closely with the Editorial Advisory Board with the aim to re-enliven our communications with this august group of experts. Closer interaction with and involvement of the Editorial Advisors can only benefit the journal and our efforts to keep it at the forefront of the computational biology community, and so we are excited about this new dimension to the journal in the months and years ahead. Finally, we are very pleased to say that Phil Bourne will continue to support the journal in an advisory capacity as Founding Editor-in-Chief—for which we are grateful.

Together, we will face the new era of Computing Biology. We will retain the open and receptive environment for all computational work which is of exceptional quality and adheres to our guidelines and principles. We hope that *PLOS Computational Biology* will provide the leadership and inspiration to make real progress. We cherish the support of the Computational Biology and broader community and will be grateful for any comments that they may have. Our mutual aim is to advance and drive exciting, productive and fruitful science.

